# Evaluation of Unity 1.5 T MR‐linac plan quality in patients with prostate cancer

**DOI:** 10.1002/acm2.14122

**Published:** 2023-08-10

**Authors:** Shohei Tanaka, Noriyuki Kadoya, Miyu Ishizawa, Yoshiyuki Katsuta, Kazuhiro Arai, Haruna Takahashi, Yushan Xiao, Noriyoshi Takahashi, Kiyokazu Sato, Ken Takeda, Keiichi Jingu

**Affiliations:** ^1^ Department of Radiation Oncology Tohoku University Graduate School of Medicine Sendai Japan; ^2^ Department of Radiological Technology School of Health Sciences Faculty of Medicine Tohoku University Sendai Japan; ^3^ Radiation Technology Tohoku University Hospital Sendai Japan

**Keywords:** IMRT, MR‐linac, prostate, radiotherapy

## Abstract

The Unity magnetic resonance (MR) linear accelerator (MRL) with MR‐guided adaptive radiotherapy (MRgART) is capable of online MRgART where images are acquired on the treatment day and the radiation treatment plan is immediately replanned and performed. We evaluated the MRgART plan quality and plan reproducibility of the Unity MRL in patients with prostate cancer. There were five low‐ or moderate‐risk and five high‐risk patients who received 36.25 Gy or 40 Gy, respectively in five fractions. All patients underwent simulation magnetic resonance imaging (MRI) and five online adaptive MRI. We created plans for 5, 7, 9, 16, and 20 beams and for 60, 100, and 150 segments. We evaluated the target and organ doses for different number of beams and segments, respectively. Variation in dose constraint between the simulation plan and online adaptive plan was measured for each patient to assess plan reproducibility. The plan quality improved with the increasing number of beams. However, the proportion of significantly improved dose constraints decreased as the number of beams increased. For some dose parameters, there were statistically significant differences between 60 and 100 segments, and 100 and 150 segments. The plan of five beams exhibited limited reproducibility. The number of segments had minimal impact on plan reproducibility, but 60 segments sometimes failed to meet dose constraints for online adaptive plan. The optimization and delivery time increased with the number of beams and segments. We do not recommend using five or fewer beams for a reproducible and high‐quality plan in the Unity MRL. In addition, many number of segments and beams may help meet dose constraints during online adaptive plan. Treatment with the Unity MRL should be performed with the appropriate number of beams and segments to achieve a good balance among plan quality, delivery time, and optimization time.

## 1 INTRODUCTION

The Elekta (Stockholm, Sweden) Unity magnetic resonance imaging‐guided linear accelerator (MRL) is widely used in a variety of tumor sites.^1^, ^2^, ^3^, ^4^, ^5^ It is a device that combines a 1.5 T magnetic resonance imaging (MRI) (Philips Healthcare, Amsterdam, Netherlands) with an accelerator equipped with a 7 MV flattening filter‐free beam. The irradiation method is step‐and‐shoot intensity‐modulated radiotherapy treatment (IMRT). The modulated dose distribution is created by combining segments of various multi‐leaf collimator shapes. Mendes et al. reported that quality of the Unity MRL plan was comparable to volumetric‐modulated arc therapy (VMAT) for conventional linear accelerator (Linac) (C‐arm Linac treatment) in terms of target coverage and organ at risk (OAR) dose reduction.^6^

The Unity MRL with MR‐guided adaptive radiotherapy (MRgART) is capable of online MRgART, in which images are acquired on the treatment day and the radiation treatment plan is immediately replanned and performed. There are two online MRgART methods: adapt to shape (ATS)—arranges the dose distributions to match organ deformations on treatment day; and adapt to position (ATP)—moves the irradiation field as the organ moves. ATP is almost identical to the treatment method utilized in image‐guided radiotherapy of conventional Linac. However, it cannot reproduce the dose distribution of simulation planning the day of treatment if the shift is >2 mm.^7^ Therefore, ATS is often used in clinical practice to benefit from the ability of MRL to capture images of soft tissues with good contrast and follow the deformation of organs.^8^, ^9^ However, this ATS requires optimization for each treatment session to change the dose distribution in accordance with patient anatomy.

Several settings are involved in this optimization process, particularly the number of beams and segments, which are considered to influence the plan quality and delivery time. Thus, these settings are very important in ensuring plan quality and reducing the amount of treatment time. In addition, a reproducible treatment plan that accommodates deformations of the prostate and rectum position, as well as bladder capacity are required for each online MRgART in patients with prostate cancer. The ability to reproduce the dose distribution of simulation planning during online treatment is important for online MRgART. Furthermore, Mannerberg et al. reported that the prostate drifts in 20% of patients after 30 min.^10^ Thus, settings with shorter optimization time and delivery time are also required to achieve the shorter overall treatment time for online MRgART. However, to the best of our knowledge, there are no reports evaluating the impact of the number of beams and number of segments on plan quality, plan reproducibility, optimization time, and delivery time in the Unity MRL.

Therefore, the aim of this study was to assess the impact of the number of beams and segments on plan quality, plan reproducibility, optimization time, and delivery time.

## 2 MATERIALS AND METHODS

### 2.1 Patient characteristics

We selected 10 patients with prostate cancer (5 low‐ or moderate‐risk and high‐risk patients, respectively) treated with MRL. All patients were treated at our hospital with 36.25 Gy (low and moderate risk) or 40 Gy (high risk) in 5 fractions. All patients had a space OAR to create space between the rectum and prostate. Table 1 shows detailed information for all patients. The study was approved by the Ethics Committee of our hospital.

**TABLE 1:** Patient characteristics in this study.

Patient	Age	Risk	PTV (cm^3^)	Prescription dose (Gy)	PTV and rectum overlap (cm^3^)	PTV and bladder overlap (cm^3^)
1	69	Low	33.1	36.25	0	2.179
2	66	Medium	89.4	36.25	0.242	4.987
3	81	Low	75.4	36.25	0.014	3.974
4	84	Low	59	36.25	0	4.128
5	72	Low	83.8	36.25	0	7.75
6	72	High	52.3	40	0.052	4.479
7	70	High	53.8	40	0	3.841
8	69	High	70.5	40	0	3.553
9	75	High	66.3	40	0.127	6.579
10	80	High	50.7	40	0	5.443

Abbreviation: PTV, planning target volume.

### 2.2 OAR and target contouring on simulation computed tomography and MRI

All patients underwent simulation CT (SOMATOM Definition AS+ system; SIEMENS) to obtain electron density information, and scanned simulation MRI (Marlin 1.5 T MRI; Philips Healthcare) to create a plan. computed tomography (CT) and MRI images were transferred to Eclipse (Version 15.6; Varian Medical Systems) and Monaco (version 5.51; Elekta), respectively. Firstly, OAR and targets were contoured on the CT image. The clinical target volume (CTV) was defined as prostate for low‐risk patients; planning target volume (PTV) added a 5 mm margin to this CTV in all directions. The CTV was defined as prostate and seminal vesicles (range: from the base to 1.5 cm from the prostate) for high‐ and moderate‐risk patients; PTV added a 5 mm margin in all directions to the CTV. Experienced radiation oncologists contoured the bladder, rectum, femoral head, and urethra as OARs. Medical physicists contoured bones for the calculation of electron density. Subsequently, we transferred this contoured CT to Monaco (Elekta). The average electron density per structure was calculated on Monaco (Elekta). We assigned electron densities to the CTV, rectum, bladder, left and right femoral head, bone, and body. Next, we transferred the structure from CT to MRI by deformable registration. Experienced radiation oncologists manually modified the transferred structure on the MRI, and final structures were completed on planning MRI.

### 2.3 OAR and target contouring on online MRgART MRI contouring

We transferred the contoured structures on the planning MRI to the online MRI by deformable registration. The radiation oncologists manually corrected the targets and OARs, and final structures were completed on online MRI. Thus, we used the structures contoured to planning MRI and contoured to online MRI for this planning study.

### 2.4 Basic settings of planning

All plans were created using the Monaco (Elekta) treatment system. The 7 MV flattening filter‐free x‐rays were used; all plans were calculated using the graphics processing unit Monte‐Carlo algorithm, a grid size of 3 mm, and an uncertainty of 1%. The detailed optimization settings were as follows: segment shape optimization was set on for all plans; high‐precision leaf positions were also set on, plan quality, minimum segment area, minimum segment width, fluence smoothing and minimum monitor units (MU)/segment was set to 5, 4 cm^2^, 0.5 cm^2^, Low, and 5.

### 2.5 Comparisons of plans for different number of beams and segments

We speculated that higher numbers of beams and segments are associated with better plan quality, but longer optimization and delivery times. In this simulation study, we compared different numbers of beams and segments to assess these effects. We evaluated the number of beams used in previous studies, that is, 5,^11^ 7,^12^, ^13^ 9,^14^, ^15^ 16,^16^, ^17^ beams. For the first time, we also examined a markedly higher number of beams (20 beams). We set the maximum segments per plan to 150 to compare the number of beams for the prevention of a decrease in plan quality due to fewer segments. Table S1 describes the gantry angle settings for each number of beams. For the comparison of number of segments, we used the maximum segments per plan (number of segments) used in previous studies (60,^18^ 100,^19^, ^20^ and 150 segments^14^). We used a uniform number of 16 beams to compare the number of segments because we considered that a smaller number of beams would limit the number of segments. We also performed a supplementary analysis using fewer beams (for example, 7) to compare the number of segments and determine whether plan quality improved by simply increasing the number of segments or by increasing the number of segments per beam. For example, if 60 segments were to be distributed over 16 beams, each beam would consist of very few segments. In this case, we hypothesized that plan quality would improve if the number of segments were to be increased with 16 beams more than increasing the number of segments with 7 beams. The results of the supplementary analysis are reported in the supplementary file.

Tables S2 and S3 show optimization parameters for low‐ or moderate‐ and high‐risk patients with prostate cancer. The optimization parameters were determined based on the dose constraints applied at our hospital. We checked the manual weights and fixed the weights of all parameters for the optimization of calculations. All plans differed only in the number of beams and segments; all other conditions remained unchanged. We created a total of 300 plans: 10 patients × 6 plans (1 simulation plan and 5 online plans) × 5 beams (5, 7, 9, 16, and 20 beams) for the number of beams. We created a total of 180 plans: 10 patients × 6 plans (1 simulation plan and 5 online plans) × 3 segments (60, 100, and 150 segments) for the number of segments.

### 2.6 Evaluation

Tables 2 and 3 show the dose constraints for low‐ and moderate‐risk and high‐risk patients, respectively. These dose constraints were applied to patients with prostate cancer treated with the Unity MRL at our hospital.

**TABLE 2:** Dose constraints for low‐ and moderate‐risk patients with prostate cancer.

Structure name		Optimal	Tolerable
PTV − rectum	D98%	>34.448 Gy	>32.625 Gy
	Dmax	<39.15 Gy	
PTV/rectum	D98%	>34 Gy	>32.625 Gy
Rectum	V36.0 Gy	<1 cc	
	V32.6 Gy	<15%	
	V29 Gy	<20%	
	V25.3 Gy	<30%	
	V21.7 Gy	<40%	
	V18.1 Gy	<50%	
Bladder	V18.1 Gy	<40%	
	V37.0 Gy	<5 cc	<10 cc
UrethraPRV	V38.0 Gy	<0.1 cc	
Femur head	V14.5 Gy	<5%	
	Dmax	<25.375 Gy	

Abbreviations: Dmax, maximal dose; D95%, dose administered to 95% of volume; D98%, dose administered to 98% of volume; PRV, planning organ at risk volume; PTV, planning target volume; PTV−Rectum, PTV minus rectum; PTV/rectum, common area of PTV and rectum.

**TABLE 3:** Dose constraints for high‐risk patients with prostate cancer.

Structure name		Optimal	Tolerable
PTV–Rectum–UrethraPRV	D98%	>34.448 Gy	>32.625 Gy
	Dmax	<43.2 Gy	
	D95%	>36.25 Gy	
PTV/rectum	D98%	>34 Gy	>32.625 Gy
Rectum	V36.0 Gy	<1 cc	
	V32.6 Gy	<15%	
	V29 Gy	<20%	
	V25.3 Gy	<30%	
	V21.7 Gy	<40%	
	V18.1 Gy	<50%	
Bladder	V18.1 Gy	<40%	
	V37.0 Gy	<5 cc	<10 cc
UrethraPRV	Dmax	<40 Gy	
Femur head	V14.5 Gy	<5%	
	Dmax	<25.375 Gy	
CTV−UrethraPRV	D95%	>100%	>98%

Abbreviations: CTV, clinical target volume; CTV−UrethraPRV, CTV minus urethraPRV; Dmax, maximal dose; D95%, dose administered to 95% of volume; D98%, dose administered to 98% of volume; PRV, planning organ at risk volume; PTV, planning target volume; PTV−Rectum−UrethraPRV, PTV minus rectum minus urethraPRV; PTV/rectum, common area of PTV and rectum.

Firstly, we evaluated the PTV and OAR doses for different number of beams and segments, respectively. We assessed statistically significant differences in dosimetry parameters between different numbers of beams and segments using the Wilcoxon signed‐rank test and MATLAB software (MathWorks, Natick, MA, USA), with a *p*‐value < 0.05 denoting statistical significance. Secondly, the variation in dose constraint between the simulation plan and online MRgART plan was measured for each patient to assess the reproducibility of the plan by changing the number of beams and segments.

Thirdly, we assessed the increase in the optimization and delivery times with the number of beams and segments. The delivery and optimization times were determined using the optimization console in offline Monaco (Elekta) treatment planning systems. Therefore, the delivery time does not reflect the actual time. Of note, it was expected that the actual delivery time would be even longer. In addition, we evaluated the MU for different numbers of beams and segments.

## 3 RESULTS

Table 4 shows the average dose parameters for target and OARs in different beam numbers (5, 7, 9, 16, 20 beams) for low‐ and high‐risk patients. For most dose constraints, there was a statistically significant difference between five and seven beams. There were also statistically significant differences for some OARs between 7 and 9 beams, 9 and 16 beams, and 16 and 20 beams. However, the proportion of significantly improved dose constraints decreased as the number of beams increased.

**TABLE 4:** Average dose parameters for the target and organ at risk with various beam numbers for low‐ or moderate‐ and high‐risk patients.

The average dose parameters for target and OAR																					
Low‐risk parietns											High‐risk parietns										
Dosimetric parameters							*p*‐value				Dosimetric parameters							*p*‐value			
		5 beams	7 beams	9 beams	16 beams	20 beams	5 beams vs. 7 beams	7 beams vs. 9 beams	9 beams vs. 16 beams	16 beams vs. 20 beams			5 beams	7 beams	9 beams	16 beams	20 beams	5 beams vs. 7 beams	7 beams vs. 9 beams	9 beams vs. 16 beams	16 beams vs. 20 beams
PTV‐Rectum‐UrethraPRV										PTV‐Rectum‐UrethraPRV											
	D98% (Gy)	35.71	35.90	35.92	35.92	35.95	<0.01	0.02	0.53	0.03		D98% (Gy)	35.26	35.37	35.39	35.45	35.43	<0.01	0.19	<0.01	0.89
	Dmax (Gy)	38.92	38.32	38.15	38.12	38.03	<0.01	<0.01	0.14	<0.01		Dmax (Gy)	43.15	42.54	42.29	42.08	42.00	<0.01	<0.01	<0.01	0.03
PTV/rectum											PTV/rectum										
	D98% (Gy)	34.73	34.63	34.89	34.74	34.61	0.79	0.05	0.73	0.85		D98% (Gy)	35.21	34.54	34.65	34.84	34.66	1.00	0.53	0.18	0.96
Rectum											Rectum										
	V36Gy (cc)	0.03	0.02	0.03	0.02	0.03	0.02	0.98	0.04	0.99		V36Gy (%)	0.08	0.05	0.07	0.06	0.05	<0.01	0.99	0.01	0.15
	V32.6 Gy (%)	1.21	1.11	1.08	1.01	1.04	0.09	0.23	0.07	0.76		V32.6 Gy (%)	0.82	0.53	0.59	0.53	0.50	<0.01	0.99	0.01	0.09
	V29Gy (%)	3.41	2.78	2.65	2.56	2.51	<0.01	0.01	0.04	0.76		V29Gy (%)	2.47	1.74	1.71	1.65	1.55	<0.01	0.49	0.14	<0.01
	V25.3 Gy (%)	6.74	4.85	4.64	4.53	4.45	<0.01	<0.01	0.05	0.16		V25.3 Gy (%)	6.26	4.47	4.05	4.10	3.93	<0.01	<0.01	0.81	0.05
	V21.7 Gy (%)	12.20	7.55	7.17	7.12	6.94	<0.01	<0.01	0.26	0.05		V21.7 Gy (%)	14.24	10.46	8.78	9.15	8.93	<0.01	<0.01	0.97	0.19
	V18.1 Gy (%)	22.44	12.20	11.24	11.69	11.21	<0.01	<0.01	0.98	0.01		V18.1 Gy (%)	29.91	24.80	19.53	20.89	20.55	<0.01	<0.01	0.99	0.27
Bladder											Bladder										
	V37Gy (cc)	2.61	1.22	0.81	0.74	0.64	<0.01	<0.01	0.03	0.10		V37Gy (%)	6.17	5.01	4.54	4.65	4.63	<0.01	<0.01	0.93	0.32
	V18.1 Gy (%)	40.81	36.72	35.73	36.29	36.35	<0.01	<0.01	0.99	0.67		V18.1 Gy (%)	40.73	36.80	33.92	36.61	36.73	<0.01	<0.01	1.00	0.69
UrethraPRV											UrethraPRV										
	V38Gy (cc)	0.03	0.00	0.00	0.00	0.00	<0.01	1.00	0.50	1.00		Dmax (Gy)	40.08	39.63	39.43	39.35	39.41	<0.01	<0.01	0.06	0.93
Left femural head											Left femural head										
	Dmax (Gy)	18.74	17.41	17.21	16.19	15.54	<0.01	0.36	<0.01	<0.01		Dmax (Gy)	19.44	15.51	18.24	14.74	13.50	<0.01	1.00	<0.01	<0.01
	V14.5 Gy (%)	5.37	2.43	2.50	1.26	1.06	<0.01	0.82	<0.01	0.10		V14.5 Gy (%)	7.05	0.33	2.49	0.15	0.00	<0.01	1.00	<0.01	<0.01
Right femural head											Right femural head										
	Dmax (Gy)	16.60	16.74	15.76	15.75	15.49	0.69	<0.01	0.65	0.04		Dmax (Gy)	16.61	15.61	16.36	14.14	12.99	<0.01	1.00	<0.01	<0.01
	V14.5 Gy (%)	2.50	1.63	0.84	2.00	1.46	0.06	<0.01	1.00	0.13		V14.5 Gy (%)	2.60	0.53	1.22	0.03	0.00	<0.01	1.00	<0.01	0.01
											CTV‐UrethraPRV										
												D95%	39.66	39.35	39.23	39.28	39.34	<0.01	<0.01	0.97	0.98

*Note*: Parameters with significant differences are indicated in red text.

Abbreviations: CTV, clinical target volume; Dmax, maximal dose; D95%, dose administered to 95% of volume; D98%, dose administered to 98% of volume; OAR, organ at risk; PRV, planning organ at risk volume; PTV, planning target volume; PTV−Rectum−Urethra, PTV minus rectum and urethraPRV structure; PTV/rectum, common areas of PTV and rectum.

Table 5 shows the average dose parameters for targets and OARs in different numbers of segments (60, 100, and 150 segments) with 16 beams for low‐ and high‐risk patients. In some dose parameters, there were statistically significant differences between 60 and 100 segments and 100 and 150 segments. In particular, the dose parameters improved due to the increase from 60 segments to 100 segments.

**TABLE 5:** Average dose parameters for the target and organ at risk using various segment numbers for low‐ and high‐risk patients.

The average dose parameters for target and OAR													
Low‐risk parietns							High‐risk parietns						
Dosimetric parameters					*p*‐value		Dosimetric parameters					*p*‐value	
		60 segments	100 segments	150 segments	60 segments vs. 100 segments	100 segments vs. 150 segments			60 segments	100 segments	150 segments	60 segments vs. 100 segments	100 segments vs. 150 segments
PTV‐Rectum							PTV‐Rectum‐Urethra						
	D98% (Gy)	35.83	35.91	35.92	<0.01	<0.01		D98% (Gy)	35.33	35.41	35.45	<0.01	<0.01
	Dmax (Gy)	38.69	38.25	38.12	<0.01	<0.01		Dmax (Gy)	42.67	42.20	42.08	<0.01	<0.01
PTV/rectum							PTV/rectum						
	D98% (Gy)	33.65	33.61	34.74	0.65	0.01		D98% (Gy)	35.17	34.90	34.84	0.96	0.65
Rectum							Rectum						
	V36Gy (cc)	0.01	0.01	0.02	0.05	0.99		V36Gy (%)	0.07	0.06	0.06	<0.01	0.62
	V32.6 Gy (%)	0.85	0.80	1.01	0.05	0.96		V32.6 Gy (%)	0.62	0.53	0.53	<0.01	0.55
	V29Gy (%)	2.40	2.31	2.56	0.06	0.97		V29Gy (%)	1.80	1.68	1.65	0.05	0.05
	V25.3 Gy (%)	4.41	4.30	4.53	0.02	0.95		V25.3 Gy (%)	4.34	4.13	4.10	0.04	0.08
	V21.7 Gy (%)	7.01	6.90	7.12	0.04	0.93		V21.7 Gy (%)	9.65	9.20	9.15	0.04	0.34
	V18.1 Gy (%)	11.40	11.36	11.69	0.32	0.99		V18.1 Gy (%)	21.13	20.63	20.89	0.17	0.86
Bladder							Bladder						
	V37Gy (cc)	1.98	1.10	0.74	<0.01	<0.01		V37Gy (%)	5.20	4.78	4.65	<0.01	0.01
	V18.1 Gy (%)	37.87	36.85	36.29	<0.01	0.01		V18.1 Gy (%)	37.54	36.67	36.61	<0.01	0.32
UrethraPRV							UrethraPRV						
	V38Gy (cc)	0.00	0.00	0.00	0.03	1.00		Dmax (Gy)	39.71	39.47	39.35	<0.01	<0.01
Left Femural Head													
	Dmax (Gy)	16.60	16.07	16.19	<0.01	0.40		Dmax (Gy)	15.21	14.74	14.74	0.01	0.56
	V14.5 Gy (%)	3.03	1.50	1.26	<0.01	0.14		V14.5 Gy (%)	0.38	0.12	0.15	0.06	0.72
Right Femural Head													
	Dmax (Gy)	16.80	15.98	15.75	<0.01	0.03		Dmax (Gy)	14.48	14.34	14.14	0.27	0.04
	V14.5 Gy (%)	4.11	2.59	2.00	<0.01	0.01		V14.5 Gy (%)	0.14	0.12	0.03	0.29	0.01
							CTV‐UrethraPRV						
								D95%	39.36	39.31	39.28	0.88	0.89

*Note*: Parameters with significant differences are indicated in red text.

Abbreviations: CTV, clinical target volume; Dmax, maximal dose; D95%, dose administered to 95% of volume; D98%, dose administered to 98% of volume; OAR, organ at risk; PRV, planning organ at risk volume; PTV, planning target volume; PTV−Rectum−Urethra, PTV minus rectum and urethra structure; PTV/rectum, common areas of PTV and rectum.

**FIGURE 1:**
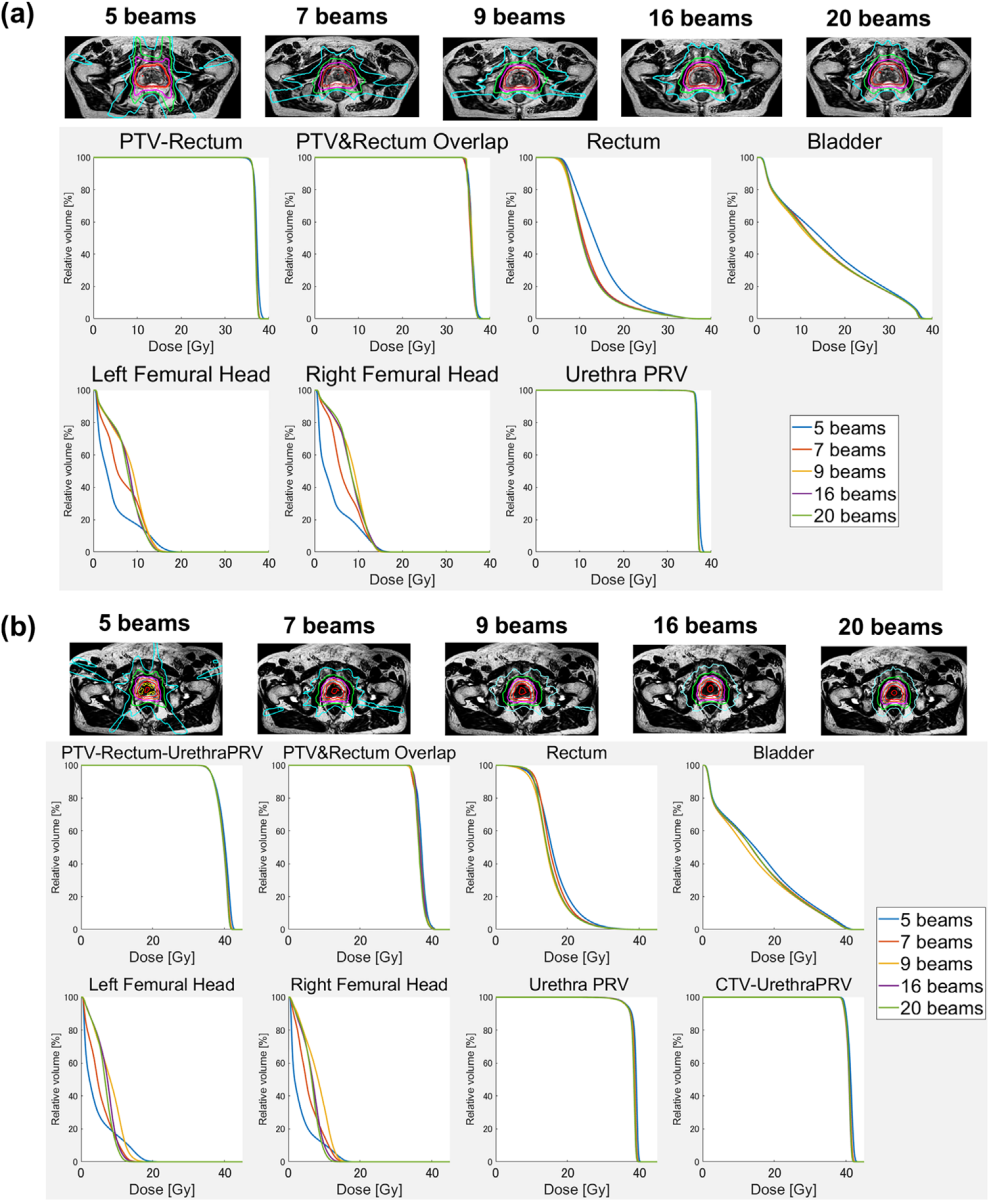
Dose distribution for typical cases and average DVH with 5, 7, 9, 16, and 20 beams for patients at low and moderate risk (a), and high risk (b). CTV, clinical target volume; DVH, dose‐volume histogram; PRV, planning organ at risk volume; PTV, planning target volume.

Figure 1a shows the dose distribution for a typical case of low‐risk patient and average dose‐volume histogram (DVH) of all low‐risk patients with regard to the number of beams. The rectum and bladder doses with five beams could not be reduced, and the maximum dose of PTV was higher compared with that of other beam numbers. However, the lower number of beams (5 or 7 beams) did not generally pass the femoral head, resulting in lower doses. There was minimal difference between 9, 16, and 20 beams for DVH curves other than the femoral head. The dose distribution in Figure 1a shows that higher numbers of beams were associated with less extension of medium and low doses into the soft tissue outside the PTV. Figure 1b shows the average DVH curves and dose distribution for high‐risk patients with regard to the number of beams. The lowest DVH of the rectum and bladder was observed with nine beams, and we did not find other major differences between patients at low and high risk.

**FIGURE 2:**
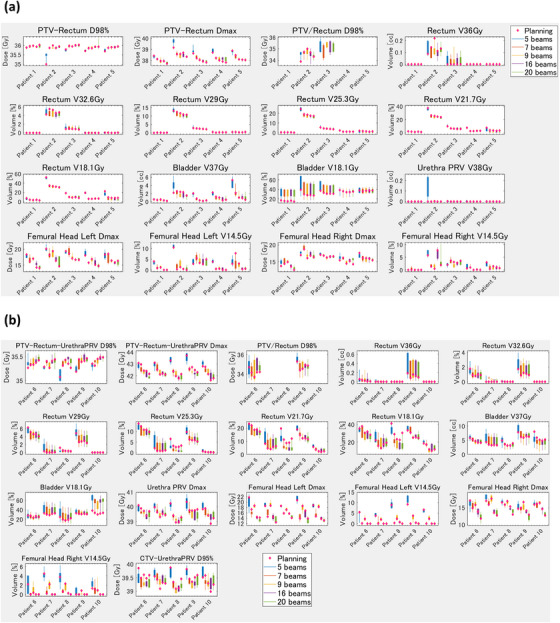
Box plot of the reproducibility of the planning and the five ART plans using various number of beams for patients at low and moderate risk (a) and high risk (b). X axis: Patient number. Y axis: The dose constraint parameters for the simulation (planning) and five online ART plans are shown in box plots. The blue, orange, yellow, purple, and green box plots are for 5, 7, 9, 16, and 20 beams respectively. The dose constraints obtained during simulation (planning) are shown as diamond plots. The shorter the box plots, the smaller the variation in the dose constraints between planning and the five online ART plans, indicating that the plan is more reproducible. On the other hand, the longer the box plots, the greater the variation in the dose constraints between planning and the five online ART plans, indicating that the plan has limited reproducibility. ART, adaptive radiotherapy; CTV, clinical target volume; Dmax, maximal dose; D95%, dose administered to 95% of volume; D98%, dose administered to 98% of volume; PRV, planning organ at risk volume; PTV, planning target volume.

Figure 2 shows the results of the robustness assessment for the various numbers of beams, low risk and high risk. Figures 2a,b show the results for low and high risk, respectively. The horizontal axis is the patient number, and the vertical axis is the dose constraint. The blue, orange, yellow, purple, and green box and whisker plots present the results for 5, 7, 9, 16, and 20 beams respectively. The values of the dose constraints (planned and for the five MRgART plans) are also depicted as box and whisker plots. The diamond plot shows the dose constraints at the time of treatment planning. Shorter box and whisker plots reveal smaller differences in dose parameters between planning and online treatment, thus indicating that the plan was robust. Overall, the box and whisker plots for five beams were longer, indicating that the plan had limited reproducibility. For seven or more beams, the box and whisker plots were minimally different in length, indicating a lack of difference in robustness.

**FIGURE 3:**
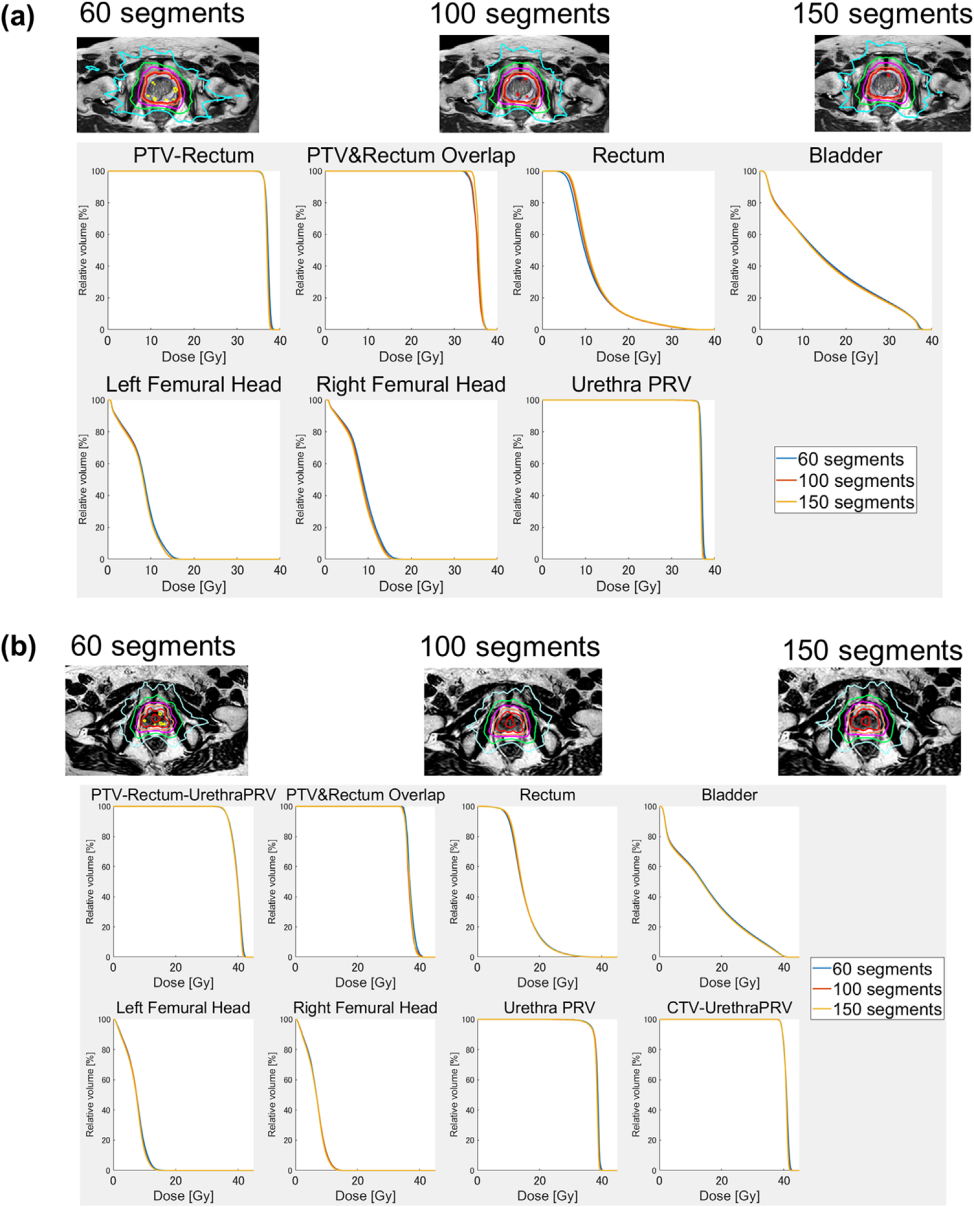
Dose distribution for typical cases and average DVH with 60, 100, and 150, segments for patients at low and moderate risk (a) and high risk (b). CTV, clinical target volume; DVH, dose‐volume histogram; PRV, planning organ at risk volume; PTV, planning target volume.

Figure 3 shows the dose distribution and average DVH curves in different segments for low‐risk patients (Figure 3a) and high‐risk patients (Figure 3b). There were no large differences in DVH and dose distribution observed between the different segments. However, the dose distribution in Figure 3 shows that higher numbers of segments were associated with less extension of medium and low doses into the soft tissue outside the PTV.

**FIGURE 4:**
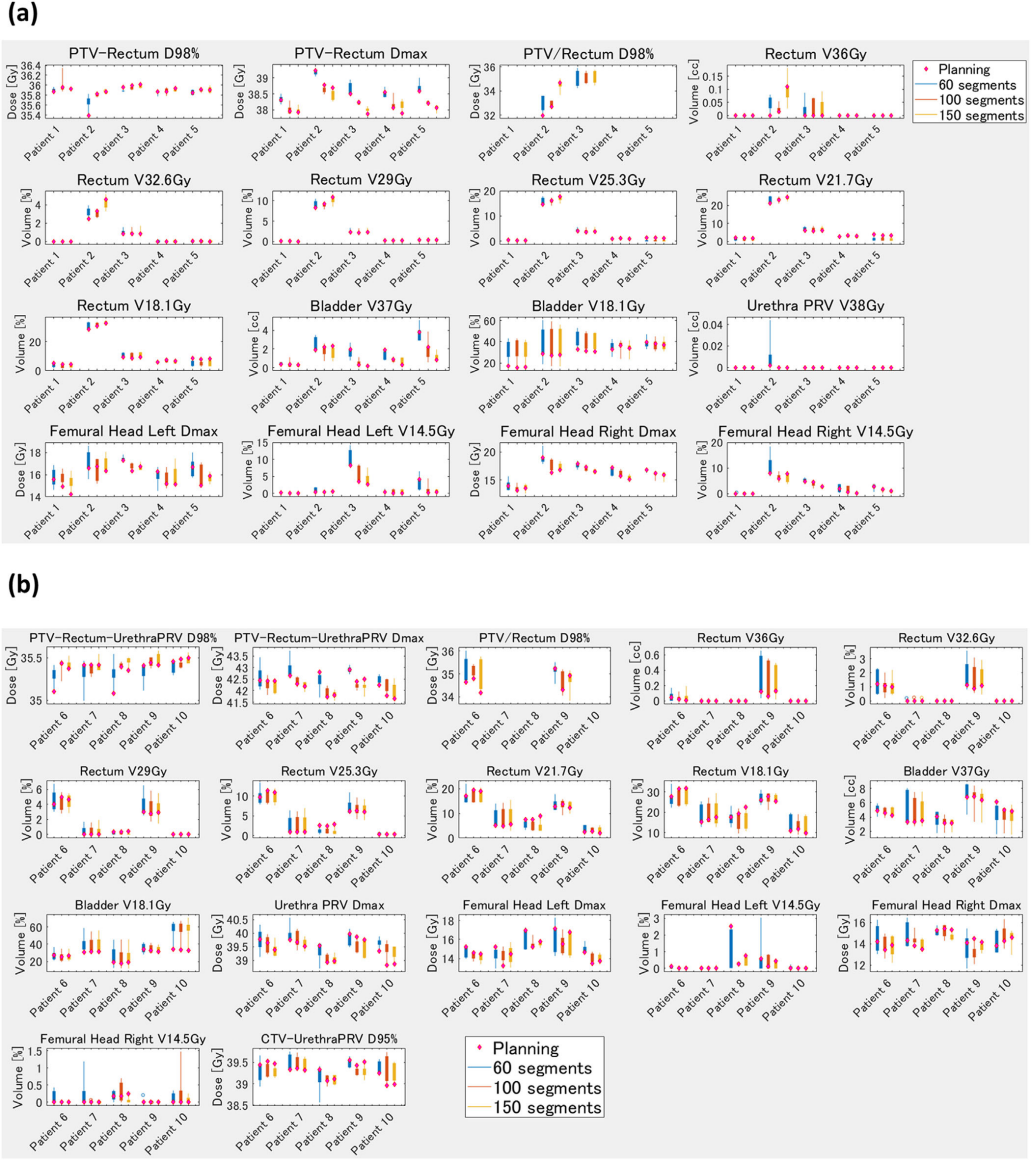
Box plot of the reproducibility of the planning and the five ART plans using varying numbers of segments for patients at low and moderate risk (a) and high risk (b). X axis: Patient number. Y axis: The dose constraint parameters for the simulation (planning) and five online ART plans are shown in box plots. The blue, orange, and yellow box plots are for 60, 100, and 150 segments respectively. The dose constraints during simulation (planning) are shown as diamond plots. The shorter the box plots, the smaller the variation in the dose constraints between the planning and the five online ART plans, indicating that the plan is more reproducible. On the other hand, the longer the box plots, the greater the variation in the dose constraint between the planning and the five online ART plans, indicating that the plan has limited reproducibility. ART, adaptive radiotherapy; CTV, clinical target volume; Dmax, maximal dose; D95%, dose administered to 95% of volume; D98%, dose administered to 98% of volume; PRV, planning organ at risk volume; PTV, planning target volume.

Figure 4 shows the results of the reproducibility assessment for the various numbers of segments and for low and high risk. The blue, orange, and yellow box and whisker plots present the results for 60, 100, and 150 beams respectively. Overall, the box and whisker plots were not markedly different in length, indicating that the number of segments had a minimal effect on the reproducibility of the plans. However, 60 segments were more variable in some dose constraints compared with 100 and 150 segments for high‐risk patients, resulting that UrethraPRV (planning organ at risk volume) Dmax and PTV–Rectum–UrethraPRV Dmax of 60 segments exceeded the dose constraint of 40 and 43.2 Gy in some patients. In addition, there was a trend toward larger box and whisker diagrams of femoral head and PTV–Rectum–UrethraPRV D98% at 60 segments for high risk compared with 100 and 150 segments.

**FIGURE 5:**
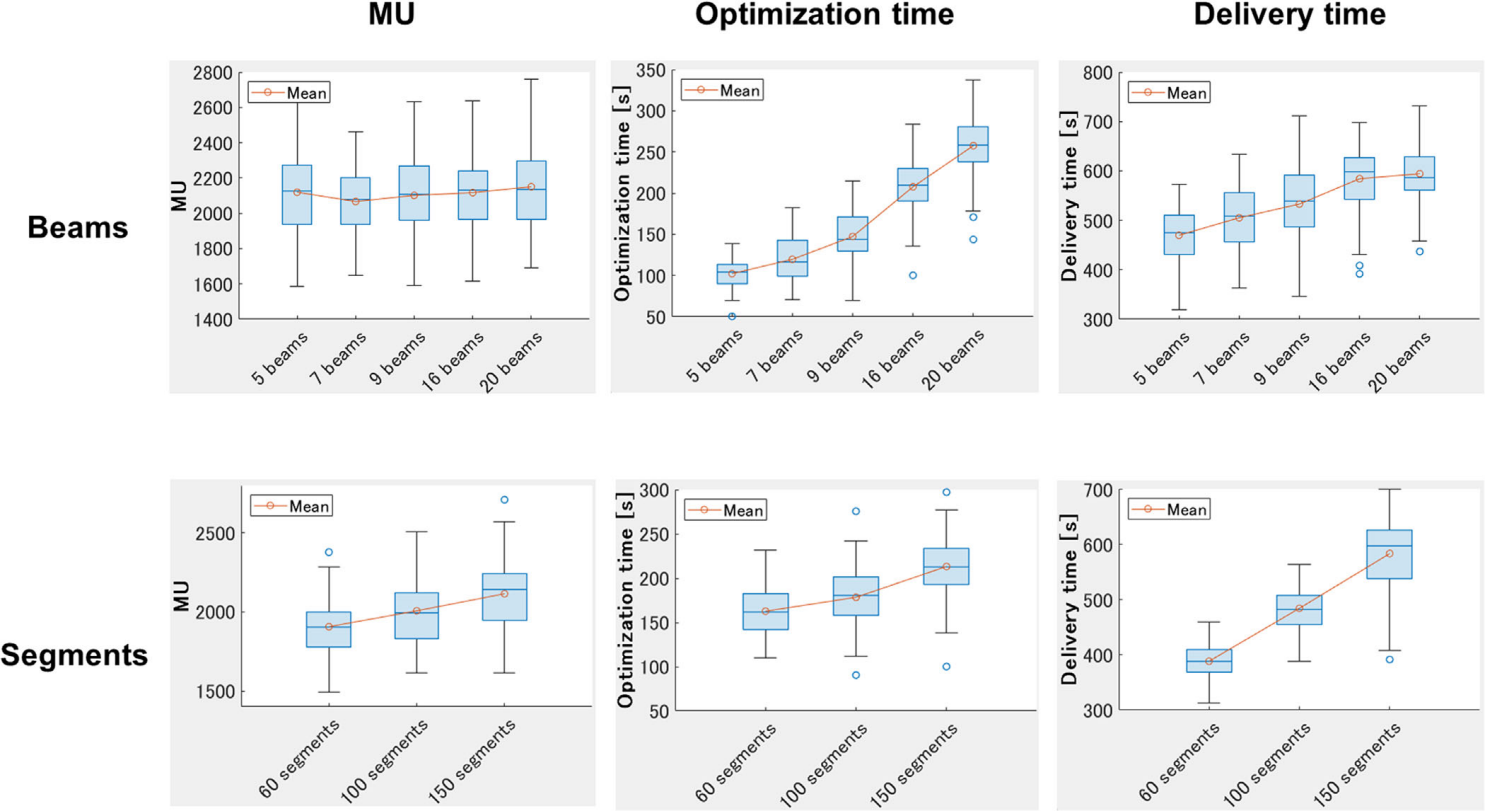
Box plots in MU, optimization time, and delivery time depending on the number of beams and segments. MU, monitor unit.

Figure 5 shows the differences in MU, optimization time, and delivery time depending on the number of beams and segments. The MU did not increase as the number of beams increased. However, the MU tended to increase in parallel with the number of segments. The optimization and delivery times tended to increase as the number of beams and segments increased.

Table S4 shows the average dose parameters for targets and OARs with varying numbers of segments (60, 100, and 150) and seven beams for low‐ and high‐risk patients. Statistically significant differences in some dose parameters were evident between 60 and 100 segments. However, no significant differences in most parameters were evident between 100 and 150 segments. The improvement in dose constraints obtained by increasing segments was therefore smaller when using seven beams than with 16 beams (Table 4 vs. Table S4). However, the MUs, delivery time, and optimization time increased with increasing segments with seven beams (Figure S1). The result was the same as that for 16 beams.

## 4 DISCUSSION

In this study, we identified significant differences for some OARs and PTV between 5 and 7, 7 and 9, 9 and 16, and 16 and 20 beams (Table 4). It has been reported that the quality of IMRT plans improves with the increasing number of beams.^21^ However, the proportion of significantly improved dose constraints decreased as the number of beams increased. Although there were no marked changes in dose constraint, the dose distribution showed a smaller extension of the medium and low doses outside the PTV as the number of beams increased (Figure 1). We thought that dose distribution might be dispersed as more beams are irradiated from various angles. Mendes et al. reported that the Unity plan was comparable to the VMAT plan.^6^ However, they also reported that VMAT achieves a better conformity index. We think that increasing the number of beams in the Unity will increase the conformity index and achieve a dose distribution similar to VMAT.

In this study, significant differences for some OARs and PTV were observed between 60 and 100 segments, and between 100 and 150 segments (Table 5). The change from 60 to 100 segments was associated with improvements in numerous dose constraints, whereas the change from 100 to 150 segments demonstrated limited improvement. In addition, the improvement in plan quality was more limited when increasing segments with 7 beams than with 16 beams (Table S4). If the 60 segments were to be distributed over 16 beams, very few segments would be allocated per beam. We, therefore, consider that the number of segments per beam is greatly improved by increasing the number of segments, thereby greatly improving the plan. However, if 60 segments are distributed over seven beams, the segments per beam are already sufficient. In this case, we consider that even if the number of segments is increased, the improvement in the number of segments per beam is small, and the improvement in plan quality is limited. In summary, when the number of beams is small, increasing the number of segments will result in only a limited improvement in plan quality. Increasing the number of segments from 100 to 150 led to increases in the MUs, delivery time, and optimization time by approximately 100 MUs, 100 s, and 35 s respectively (Figure 5). Keizer et al. reported that, for some patients, the prostate moved by approximately 2 mm in the caudal and cranial direction within 3 min^22^; thus, we consider that an increase in the number of segments is both unnecessary and unacceptable, particularly when the number of beams is small.

Use of five beams was associated with large variation in the reproducibility of the plan; however, the use of seven or more beams was linked to minimal variation (Figure 2). Due to variation in the plan quality for the Unity MRL, numerous studies reported that they could not meet the dose constraints in online MRgART. McDonald et al. reported that an adaptive plan failed to meet dose constraints several times during treatment for most patients with head and neck cancer.^23^ In addition, Werensteijn‐Honingh et al.^24^ and Henke et al.^25^ also reported that an adaptive plan failed to meet the OAR constraints. Although dose constraints are met during simulation planning, they are occasionally not met in the online MRgART due to organ deformations or statistical uncertainties of the dose calculation in the Unity MRL. This requires reproducible planning that can meet the dose constraints for online MRgART. Figure 2 shows that there was a large variation in dose constraint for five beams, while there was no large difference in the variation for seven beams and more. Thus, we do not recommend five beams or fewer for online MRgART, and at least seven beams are necessary for reproducible planning in Unity. In addition, Figure 4 shows that 60 segments were more variable in some dose constraints compared with 100 and 150 segments for high‐risk patients. Plans with more segments may be preferable than those with fewer segments for patients who have difficulty in meeting dose constraints. Furthermore, we considered the effect of PTV on patient‐specific dose parameters. In general, we consider that patients with a larger PTV require a more complex plan because of overlap with the rectum and bladder and closer distances to the femoral head. Among the low‐risk patients, the PTV was smaller for patients 1 and 4, and larger for patients 2 and 5 (Table 1). Patient 2 had an overlap with the rectum; thus, the rectum dose was improved by increasing the number of beams (Figure 2a). Patient no. 5 had a large overlap with the bladder (Table 1), and so the bladder V37 Gy was improved by increasing the number of beams. With fewer beams (5), the box and whisker plots for those two patients were longer, indicating poor reproducibility. On the other hand, patients 1 and 4 with their smaller PTVs had no overlap with the rectum and a small overlap with the bladder (Table 1 ), in a manner where the improvement in dose parameters for the rectum and bladder was small when the number of beams was increased (Figure 2a). Among the high‐risk patients, patients 6 and 7 had smaller PTVs, and patients 8 and 9 had larger PTVs (Table 1). In particular, the overlap between the PTV and both the rectum and bladder were large in patient 9, and the doses to those OARs improved as the number of beams increased (Figure 2b). In summary, rectum and bladder doses improved when the number of beams was increased for patients with a large PTV and large overlaps with the rectum and bladder. On the other hand, in comparing the number of segments, only a limited improvement in the rectum dose was obtained by increasing the number of segments for patients 2 and 9, who had large PTVs and large overlaps with the rectum (Figure 4). Increasing the number of segments might improve PTV coverage and the maximum dose to the PTV and the urethraPRV; however, reducing the low and medium dose to the rectum and bladder might be difficult (Figure 4).

In this study, we observed that the optimization and delivery times increased in parallel with the number of beams and segments (Figure 5). Mittauer et al. reported that a larger minimum MU parameter leads to a smaller number of segments and shorter delivery time in Elekta Linac.^26^ In other words, they showed a tendency for decrease in delivery time in parallel with the number of segments. Quan et al.^21^ also reported that the delivery time usually increased in parallel with the number of beams in Linac, which is consistent with the findings of the present study. Online MRgART with the Unity MRL requires longer treatment time (i.e., approximately 50 min).^27^ If an organ transfer occurs during online MRgART, the optimization time should ideally be short, because the process is reinitiated from the first fusion. In addition, the dose delivery method utilized in the Unity MRL is step‐and‐shoot, and the delivery time is long (approximately 11 min).^28^ Some patients move approximately 2 mm in the caudal and cranial direction within the first 3 min from beam activation in the treatment of prostate cancer using the Unity MRL^22^; shorter delivery times are ideal for such patients. Therefore, the risk of prostate movement can be minimized by reducing the number of beams and segments. Currently, Unity MRL only includes an IMRT method; however, it will also include the VMAT method in the future.^29^ If VMAT becomes possible, the delivery time could be significantly shorter and these problems might be solved.

A limitation of this study is that we only used one template for planning. We determined this plan template based on the clinical protocol of our hospital. This plan template did not have a high complexity index to reproduce the simulation plan in online MRgART. Therefore, it is likely that there was no marked difference in dose distribution and plan reproducibility between 9, 16, and 20 beams. If we had used a plan template with a higher complexity index that could significantly reduce the rectal and bladder doses, the improvement in dose constraint due to the increased number of beams may have been more pronounced. Further investigation is warranted to test this hypothesis.

## 5 CONCLUSION

This study evaluated the quality, reproducibility, delivery time, and optimization time of the Unity MRL plans for different number of beams and segments. Plans with few beams exhibited significantly lower plan quality and reproducibility than plans with a higher number of beams. Increasing the number of beams improved the plan quality. Moreover, increasing the number of segments slightly improved plan quality and reproducibility. However, increasing the number of beams and segments also extended the delivery and optimization times. Treatment with the Unity MRL should be performed with the appropriate number of beams and segments to achieve a good balance among plan quality, delivery time, and optimization time.

## AUTHOR CONTRIBUTIONS

Shohei Tanaka, Noriyuki Kadoya, Yoshiyuki Katsuta, Kazuhiro Arai, Haruna Takahashi, Yushan Xiao, Noriyoshi Takahashi, and Kiyokazu Sato contributed to the plan and idea of the study. Shohei Tanaka, Noriyuki Kadoya, and Miyu Ishizawa performed the analysis. Shohei Tanaka mainly drafted the manuscript. Yoshiyuki Katsuta, Kazuhiro Arai, Haruna Takahashi, Yushan Xiao, Noriyoshi Takahashi, and Kiyokazu Sato provided guidance regarding planning. Noriyoshi Takahashi and Ken Takeda provided clinical knowledge. Noriyuki Kadoya and Keiichi Jingu reviewed the manuscript. All authors read and approved the final manuscript.

## CONFLICT OF INTEREST STATEMENT

Dr Keiichi Jingu has rewarded a research grant from Elekta.

## Supporting information

Supporting Information

## References

[1] Mönnich D , Winter J , Nachbar M , et al. Quality assurance of IMRT treatment plans for a 1.5 T MR‐linac using a 2D ionization chamber array and a static solid phantom. Phys Med Biol. 2020;65(16):16nt01. 10.1088/1361-6560/aba5ec 32663819

[2] Tyagi N , Liang J , Burleson S , et al. Feasibility of ablative SBRT treatment of pancreas patients on an MR‐Linac. Int J Radiat Oncol Biol Phys. 2021;111(3):e557.

[3] Janssen TM , Aitken K , Alongi F , et al. First multicentre experience of SABR for lymph node and liver oligometastatic disease on the unity MR‐Linac. Tech Innov Patient Support Radiat Oncol. 2022;22:50‐54. 35586786 10.1016/j.tipsro.2022.04.005 PMC9108982

[4] Gani C , Boeke S , McNair H , et al. Marker‐less online MR‐guided stereotactic body radiotherapy of liver metastases at a 1.5 T MR‐Linac–Feasibility, workflow data and patient acceptance. Clin Transl Radiat Oncol. 2021;26:55‐61. 33319073 10.1016/j.ctro.2020.11.014 PMC7723999

[5] Dunlop A , Mitchell A , Tree A , et al. Daily adaptive radiotherapy for patients with prostate cancer using a high field MR‐linac: initial clinical experiences and assessment of delivered doses compared to a C‐arm linac. Clin Transl Radiat Oncol. 2020;23:35‐42. 32395640 10.1016/j.ctro.2020.04.011 PMC7210377

[6] Da Silva Mendes V , Nierer L , Li M , et al. Dosimetric comparison of MR‐linac‐based IMRT and conventional VMAT treatment plans for prostate cancer. Radiat Oncol. 2021;16(1):133. 34289868 10.1186/s13014-021-01858-7 PMC8296626

[7] Gupta A , Dunlop A , Mitchell A , et al. Online adaptive radiotherapy for head and neck cancers on the MR linear accelerator: introducing a novel modified adapt‐to‐shape approach. Clin Transl Radiat Oncol. 2022;32:48‐51. 34849412 10.1016/j.ctro.2021.11.001 PMC8608651

[8] Alongi F , Rigo M , Figlia V , et al. 1.5 T MR‐guided and daily adapted SBRT for prostate cancer: feasibility, preliminary clinical tolerability, quality of life and patient‐reported outcomes during treatment. Radiat Oncol. 2020;15:1‐9. 10.1186/s13014-020-01510-w PMC7092497 32248826

[9] Intven MP , van Otterloo SD , Mook S , et al. Online adaptive MR‐guided radiotherapy for rectal cancer; feasibility of the workflow on a 1.5 T MR‐linac: clinical implementation and initial experience. Radiother Oncol. 2021;154:172‐178. 32976875 10.1016/j.radonc.2020.09.024

[10] Mannerberg A , Persson E , Jonsson J , et al. Dosimetric effects of adaptive prostate cancer radiotherapy in an MR‐linac workflow. Radiat Oncol. 2020;15:1‐9. 10.1186/s13014-020-01604-5 PMC7350593 32650811

[11] Kontaxis C , de Muinck Keizer DM , Kerkmeijer LG , et al. Delivered dose quantification in prostate radiotherapy using online 3D cine imaging and treatment log files on a combined 1.5 T magnetic resonance imaging and linear accelerator system. Phys Imaging Radiat Oncol. 2020;15:23‐29. 33458322 10.1016/j.phro.2020.06.005 PMC7807644

[12] de Muinck Keizer DM , de Groot‐van Breugel EN , Raaymakers BW , Lagendijk JJ , de Boer HC . On‐line daily plan optimization combined with a virtual couch shift procedure to address intrafraction motion in prostate magnetic resonance guided radiotherapy. Phys Imaging Radiat Oncol. 2021;19:90‐95. 34377842 10.1016/j.phro.2021.07.010 PMC8327343

[13] Axford A , Dikaios N , Roberts DA , Clark CH , Evans PM . An end‐to‐end assessment on the accuracy of adaptive radiotherapy in an MR‐linac. Phys Med Biol.2021;66(5):055021. 10.1088/1361-6560/abe053 33503604

[14] Willigenburg T , Beld E , Hes J , Lagendijk JJ , de Boer HC , Moerland MA . Focal salvage treatment for radiorecurrent prostate cancer: a magnetic resonance‐guided stereotactic body radiotherapy versus high‐dose‐rate brachytherapy planning study. Phys Imaging Radiat Oncol. 2020;15:60‐65. 33458327 10.1016/j.phro.2020.07.006 PMC7807590

[15] Mohajer J , Dunlop A , Mitchell A , et al. Feasibility of MR‐guided ultrahypofractionated radiotherapy in 5, 2 or 1 fractions for prostate cancer. Clin Transl Radiat Oncol. 2021;26:1‐7. 33241129 10.1016/j.ctro.2020.10.005 PMC7674276

[16] Alongi F , Rigo M , Figlia V , et al. Rectal spacer hydrogel in 1.5 T MR‐guided and daily adapted SBRT for prostate cancer: dosimetric analysis and preliminary patient‐reported outcomes. Br J Radiol. 2021;94(1117):20200848. 33095659 10.1259/bjr.20200848 PMC7774687

[17] Künzel LA , Nachbar M , Hagmüller M , et al. First experience of autonomous, un‐supervised treatment planning integrated in adaptive MR‐guided radiotherapy and delivered to a patient with prostate cancer. Radiother Oncol. 2021;159:197‐201. 33812912 10.1016/j.radonc.2021.03.032

[18] Bertelsen AS , Schytte T , Møller PK , et al. First clinical experiences with a high field 1.5 T MR linac. Acta Oncol. 2019;58(10):1352‐1357. 31241387 10.1080/0284186X.2019.1627417

[19] Ruggieri R , Rigo M , Naccarato S , et al. Adaptive SBRT by 1.5 T MR‐linac for prostate cancer: on the accuracy of dose delivery in view of the prolonged session time. Phys Med. 2020;80:34‐41. 33091796 10.1016/j.ejmp.2020.09.026

[20] Naccarato S , Rigo M , Pellegrini R , et al. Automated planning for prostate stereotactic body radiation therapy on the 1.5 T MR‐Linac. Adv Radiat Oncol. 2022;7(3):100865. 35198836 10.1016/j.adro.2021.100865 PMC8850203

[21] Quan EM , Li X , Li Y , et al. A comprehensive comparison of IMRT and VMAT plan quality for prostate cancer treatment. Int J Radiat Oncol Biol Phys. 2012;83(4):1169‐1178. 22704703 10.1016/j.ijrobp.2011.09.015 PMC3805837

[22] de Muinck Keizer D , Kerkmeijer L , Willigenburg T , et al. Prostate intrafraction motion during the preparation and delivery of MR‐guided radiotherapy sessions on a 1.5 T MR‐Linac. Radiother Oncol. 2020;151:88‐94. 32622779 10.1016/j.radonc.2020.06.044

[23] McDonald BA , Vedam S , Yang J , et al. Initial feasibility and clinical implementation of daily mr‐guided adaptive head and neck cancer radiation therapy on a 1.5 t mr‐linac system: prospective r‐ideal 2a/2b systematic clinical evaluation of technical innovation. Int J Radiat Oncol Biol Phys. 2021;109(5):1606‐1618. 33340604 10.1016/j.ijrobp.2020.12.015 PMC7965360

[24] Werensteijn‐Honingh AM , Kroon PS , Winkel D , et al. Impact of magnetic resonance‐guided versus conventional radiotherapy workflows on organ at risk doses in stereotactic body radiotherapy for lymph node oligometastases. Phys Imaging Radiat Oncol. 2022;23:66‐73. 35814260 10.1016/j.phro.2022.06.011 PMC9263510

[25] Henke LE , Olsen JR , Contreras JA , et al. Stereotactic MR‐guided online adaptive radiation therapy (SMART) for ultracentral thorax malignancies: results of a phase 1 trial. Adv Radiat Oncol. 2019;4(1):201‐209. 30706029 10.1016/j.adro.2018.10.003 PMC6349650

[26] Mittauer K , Lu B , Yan G , et al. A study of IMRT planning parameters on planning efficiency, delivery efficiency, and plan quality. Med Phys. 2013;40(6):061704. 23718583 10.1118/1.4803460

[27] Cuccia F , Mazzola R , Nicosia L , et al. Impact of hydrogel peri‐rectal spacer insertion on prostate gland intra‐fraction motion during 1.5 T MR‐guided stereotactic body radiotherapy. Radiat Oncol. 2020;15(1):1‐9. 10.1186/s13014-020-01622-3 PMC7376654 32698843

[28] Willigenburg T , Zachiu C , Bol GH , et al. Clinical application of a sub‐fractionation workflow for intrafraction re‐planning during prostate radiotherapy treatment on a 1.5 Tesla MR‐Linac: a practical method to mitigate intrafraction motion. Radiother Oncol. 2022;176:25‐30. 36113777 10.1016/j.radonc.2022.09.004

[29] Uijtewaal P , Borman PT , Woodhead PL , et al. First experimental demonstration of VMAT combined with MLC tracking for single and multi fraction lung SBRT on an MR‐linac. Radiother Oncol. 2022;174:149‐157. 35817325 10.1016/j.radonc.2022.07.004

