# Supplementary material for: Evaluation of Unity 1.5 T MR‐linac plan quality in patients with prostate cancer
Source: J Appl Clin Med Phys. 2023 Aug 10;24(12):e14122. doi: 10.1002/acm2.14122 (PMC10691646; doi:10.1002/acm2.14122)
Supplement: Supplementary file 1 — Supporting Information [file ACM2-24-e14122-s001.pdf]

SUPPLEMENTARY INFORMATION

Evaluation of Unity 1.5 T MR-Linac plan quality in patients with prostate cancer

Shohei Tanaka[1], Noriyuki Kadoya[1], Miyu Ishizawa[2], Yoshiyuki Katsuta[1], Kazuhiro Arai[1], Haruna Takahashi[1], Yushan Xiao[1], Noriyoshi Takahashi[1], Kiyokazu Sato[3], Ken Takeda[2], Keiichi Jingu[1]

[1]Department of Radiation Oncology, Tohoku University Graduate School of Medicine, Sendai, Japan

[2]Department of Radiological Technology, School of Health Sciences, Faculty of Medicine, Tohoku University, Sendai, Japan

[3]Radiation Technology, Tohoku University Hospital, Sendai, Japan

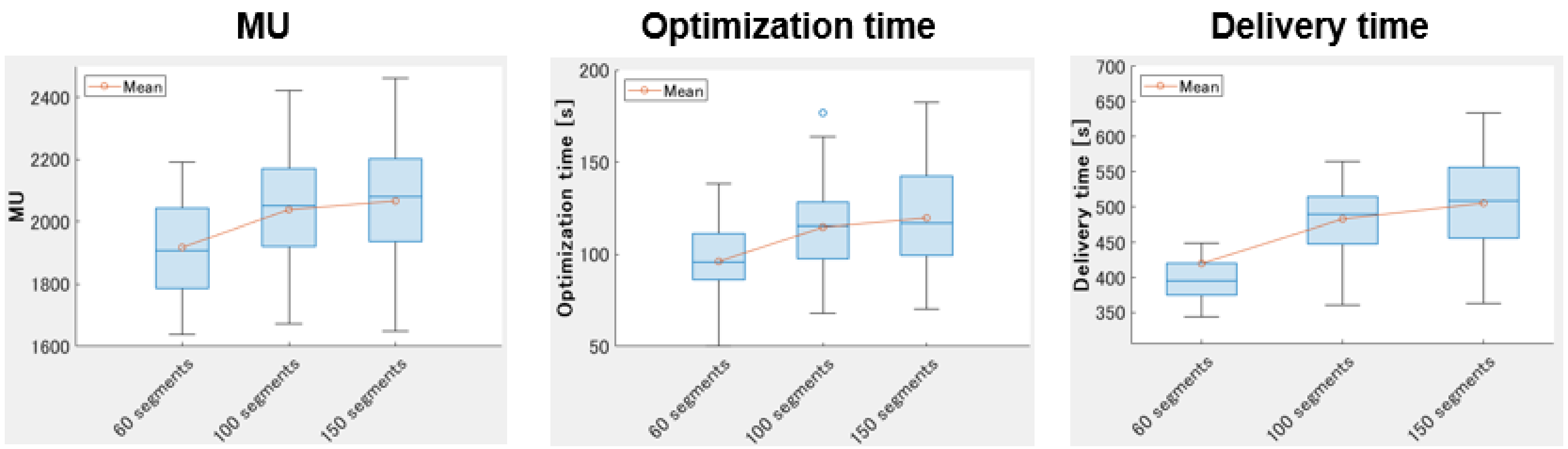

FIGURE S1 Box plots for monitor units (MU), optimization time, and delivery time achieved using various segment numbers with 7 beams.

TABLE S1 Gantry angle settings for each number of beams

Number of beams	Gantry angle (°)
5	0, 72, 144, 216, 288
7	0, 51, 102, 153, 204, 255, 306
9	0, 50, 75, 100, 150, 210, 260, 285, 310
16	5, 25, 45, 65, 85, 105, 136, 157, 203, 224, 255, 275, 295, 315, 335, 355
20	6, 24, 42, 60, 78, 96, 114, 136, 153, 170, 190, 207, 224, 246, 263, 280, 297, 314, 331, 348

TABLE S2 Optimization parameters for low-risk and moderate-risk patients

Structure name	Cost function	Manual	Weight	Minimum volume (%)	Reference dose (Gy)	Power law exponent	Shrink margin (cm)	Isoconstraint
PTV−Rectum	Target penalty	✓	3	99				36.5
	Quadratic overdose	✓	1		37			1
	Quadratic overdose	✓	2		38			0.02
PTV/Rectum	Target penalty	✓	1	99.9				34.5
Rectum	Quadratic overdose	✓	0.2		35.1		0	0.2
	Serial	✓	1			12	0.3	24

	Serial	✓	5		10	0.6	14
	Quadratic overdose	✓	0.5	36.8			0.04
Bladder	Quadratic overdose	✓	0.5	32.63		0	0.4
	Serial	✓	2		10	0.6	17
Left femoral head	Quadratic overdose	✓	0.3	14		0	0.1
Right femoral head	Quadratic overdose	✓	0.3	14		0	0.1
Urethra PRV	Quadratic overdose	✓	0.3	37.5			0.02

Body	Quadratic overdose	✓	0.5	36.25	0.1	0.02
	Quadratic overdose	✓	0.3	18.17	1.5	0.5

PRV, planning organ at risk volume; PTV, planning target volume; PTV/Rectum, overlap between PTV and rectum.

TABLE S3 Optimization parameters for high-risk patients

Structure name	Cost function	Manual	Weight	Minimum volume (%)	Reference dose (Gy)	Power law exponent	Shrink margin (cm)	Isoconstraint
Urethra PRV	Quadratic overdose	✓	1		39.2			0.02
CTV	Target penalty	✓	1.5	99.5				41
PTV−Rectum−Urethra PRV	Target penalty	✓	2	95				36.3
	Quadratic overdose	✓	3		42			0.02
	Target penalty	✓	1	99				34.4
PTV/Rectum	Target penalty	✓	1	99.9				34.5
Rectum	Quadratic	✓	0.5		36		0	0.1

	overdose						
	Serial	✓	1		12	0.3	24
	Serial	✓	5		10	0.6	14
	Quadratic overdose	✓	0.3	39		0.1	0.05
Bladder	Quadratic overdose	✓	0.5	36		0.1	0.1
	Serial	✓	2		10	0.6	17
Left femoral head	Quadratic overdose	✓	0.3	14		0	0.1
Right femoral head	Quadratic overdose	✓	0.3	14		0	0.1

Prostate + 1.5 cm	Quadratic overdose	✓	0.5	36.25	0.1	0.1
Body	Quadratic overdose	✓	0.3	18.75	1.5	0.3

CTV, clinical target volume; PRV, planning organ at risk volume; PTV, planning target volume; PTV/Rectum, overlap between PTV and rectum.

TABLE S4 Average dose parameters for the target and organ at risk (OAR) using various segment numbers with 7 beams for low- and high-risk patients

The average dose parameters for target and OAR											
Low-risk patients						High-risk patients					
Dosimetric parameters				p value		Dosimetric parameters				p value	
	60 segments	100 segments	150 segments	60 segments vs. 100 segments	100 segments vs. 150 segments		60 segments	100 segments	150 segments	60 segments vs. 100 segments	100 segments vs. 150 segments
PTV-Rectum						PTV-Rectum-Urethra					
D98% (Gy)	35.87	35.88	35.90	0.26	0.01	D98% (Gy)	35.33	35.39	35.37	< 0.01	0.99
Dmax (Gy)	38.60	38.35	38.32	< 0.01	0.14	Dmax (Gy)	42.77	42.52	42.54	< 0.01	0.67
PTV/Rectum						PTV/Rectum					
D98% (Gy)	34.82	34.44	34.63	0.99	0.18	D98% (Gy)	34.83	34.67	34.54	0.72	0.35
Rectum						Rectum					
V36Gy (cc)	0.04	0.03	0.02	0.02	0.32	V36Gy (%)	0.06	0.05	0.05	0.01	0.96
V32.6Gy (%)	1.25	1.15	1.11	< 0.01	0.06	V32.6Gy (%)	0.60	0.54	0.53	0.01	0.45
V29Gy (%)	2.93	2.84	2.78	0.15	0.14	V29Gy (%)	1.85	1.77	1.74	0.12	0.19
V25.3Gy (%)	4.97	4.93	4.85	0.21	0.48	V25.3Gy (%)	4.58	4.48	4.47	0.16	0.24
V21.7Gy (%)	7.65	7.69	7.55	0.37	0.12	V21.7Gy (%)	10.48	10.41	10.46	0.40	0.36
V18.1Gy (%)	12.18	12.37	12.20	0.79	0.19	V18.1Gy (%)	24.40	24.59	24.80	0.78	0.79
Bladder						Bladder					
V37Gy (cc)	1.80	1.21	1.22	< 0.01	0.52	V37Gy (%)	5.17	4.97	5.01	0.03	0.79
V18.1Gy (%)	37.40	36.86	36.72	0.01	0.13	V18.1Gy (%)	36.82	36.73	36.80	0.26	0.74
Urethra PRV						Urethra PRV					
V38Gy (cc)	0.00	0.00	0.00	0.25	0.50	Dmax (Gy)	39.79	39.58	39.63	< 0.01	0.88
Left Femural Head											
Dmax (Gy)	17.87	17.44	17.41	0.03	0.48	Dmax (Gy)	15.41	15.27	15.51	0.31	0.85
V14.5Gy (%)	3.61	2.66	2.43	0.02	0.34	V14.5Gy (%)	0.64	0.31	0.33	0.11	0.64
Right Femural Head											
Dmax (Gy)	16.97	16.70	16.74	0.04	0.64	Dmax (Gy)	15.76	15.61	15.61	0.07	0.54
V14.5Gy (%)	1.98	1.66	1.63	0.11	0.39	V14.5Gy (%)	0.77	0.56	0.53	0.17	0.17
						CTV-Urethra PRV					
						D95%	39.39	39.30	39.35	0.99	0.06

CTV, clinical target volume; Dmax, maximal dose; D95%, dose administered to 95% of volume; D98%, dose administered to 98% of volume; OAR, organ at risk; PRV, planning organ at risk volume; PTV, planning target volume; PTV−Rectum−Urethra, PTV minus rectum and urethra structure; PTV/Rectum, common areas of PTV and rectum.
